# Prevalence of non-communicable diseases (NCDs) and associated factors among HIV positive educators: Findings from the 2015/6 survey of Health of Educators in Public Schools in South Africa

**DOI:** 10.1371/journal.pone.0209756

**Published:** 2019-02-08

**Authors:** Nompumelelo Precious Zungu, Musawenkosi Lionel Mabaso, Faith Kumalo, Salome Sigida, Lungelo Mlangeni, Njeri Wabiri, Charles Chasela

**Affiliations:** 1 Human Sciences Research Council, Pretoria, South Africa; 2 Department of Psychology, University of Pretoria, Pretoria, South Africa; 3 Department of Basic Education, Pretoria, South Africa; 4 EQUIP Programme, Right to Care, Johannesburg, South Africa; 5 Department of Epidemiology and Biostatistics, School of Public Health, Faculty of Health Sciences, University of the Witwatersrand, Johannesburg, South Africa; University of California, UNITED STATES

## Abstract

**Introduction:**

In many sub-Saharan African countries, confronting the dual epidemic of HIV and NCDs is a public health priority especially in high HIV burden countries such as South Africa. Evidence shows that poor health as a consequence of NCDs and HIV among the workforce increases absenteeism and leads to decrease in productivity. However, the prevalence of these co-occurring chronic conditions and associated factors is unknown in the educator workforce. Improved understanding has implications for their management and wellbeing of educators. This paper reports the prevalence of selected NCDs and associated factors among HIV positive educators in South Africa using the 2015/6 survey of Educators in Public Schools in South Africa.

**Methods:**

This was a second-generation surveillance undertaken among educators in selected public schools in all nine provinces in South Africa. A multi-stage stratified cluster design with probability proportional to size sampling was used to draw a random sample of schools. Factors associated with presence of NCDs were determined using a multivariate backward stepwise logistic regression analysis.

**Results:**

A total of 1 365 schools were sampled within which 21 495 (85.5%) educators were interviewed. Out of 2691, HIV Positive educators that responded to the questions on NCDs, 36.9% reported having NCDs. The most commonly reported NCDs were high blood pressure (17.4%), and stomach ulcers (13.5%). The increased odds of reporting the presence of NCDs was significantly associated with being female than male [aOR = 1.5: 95% CI (1.1–1.9), p<0.002], age 45 to 54 years [aOR = 1.8: 95% CI (1.4–2.2), p = p<0.001], and age 55 years and older than those 18 to 24 years [aOR = 2.7: 95% CI (1.8–3.9), p<0.001). The decreased odds of reporting the presence of NCDs was significantly associated with not being absent from school for health reasons [aOR = 0.7: 95% CI (0.6–0.9), p = 0.003].

**Conclusion:**

NCDs care and active screening should be an integral part of HIV programmes including interventions such as prevention, treatment, care and support amongst public school educators in SA. The education department will need to invest in health promotion intervention programmes to prevent and mitigate the negative impact of NCDs and HIV on the sector.

## Introduction

In sub-Saharan Africa, non-Communicable Diseases (NCDs) in the context of HIV and antiretroviral provision are an emerging public health challenge especially in high HIV burden populations like South Africa [1–7]. As in many sub-Saharan African countries, confronting the dual epidemic of HIV and NCDs is a public health priority. The country has the highest number of people living with HIV (PLHIV) in the world [8], while concurrently the prevalence of NCDs is high and accounts for high morbidity and mortality [1,6,7,9]

Like HIV, NCDs disproportionately affect poor people. In South Africa, this is intertwined with rapid urbanization and unplanned demographic shift leading to risky and unhealthy life styles in the era of HIV [6,10]. Addressing the comorbid non-communicable disease (NCD) and HIV epidemics is important for improved public health outcomes and better economic growth in the country [11,12]. Commonly reported NCDs among PLHIV include cardiovascular diseases, diabetes, cancers, chronic pulmonary diseases, liver disease, chronic kidney disease, hypertension and depression [6–8].

The determinants of NCD comorbidities in HIV/AIDS vary based on the specific NCD considered. Generally, increased age and increased immune suppression, overweight and obesity, social deprivation, and longer duration of exposure to antiretroviral treatments are some of the most common NCD risk factors in HIV infected individuals [6,8]. Unless urgent action is taken against the rising NCD burden among PLHIV, NCDs will add great pressure to the already existing challenge in the fight against HIV [11,12].

Evidence shows that poor health as a consequence of NCDs and HIV among the workforce increase absenteeism and leads to decrease in productivity [13,14]. However, not much is known about the prevalence and risk factors associated with NCDs among HIV positive workforce in the country. This paper reports the prevalence of NCDs and associated factors among HIV positive educators in South Africa using the 2015/6 survey of Educators in Public Schools in South Africa [15]. Understanding prevalence of these co-occurring chronic conditions and associated factors has implications for their management and the wellbeing of educators, which is a critical factor in the planning and resourcing of the education sector in order to achieve quality outcomes.

## Methodology

### Study design and sample

This study used data collected as part of the 2015/6 national educator’s survey in South Africa; described in detail elsewhere [15]. The survey followed a cross-sectional approach employing second-generation surveillance methods that combined collection of socio-demographic, behavioural data and a blood specimen using a Dried Blood Spot (DBS) collection. The Education Management Information System (EMIS) Master List of educators from the Department of Basic Education (DBE) for 2012 was used as the sampling frame to identify the schools and number of educators at each school. This comprised 25,179 schools with 389,044 educators. HIV prevalence among educators in South Africa was estimated at 12.7% [16]. Assuming an HIV testing response rate of 73%, a minimum sample size of 27,869 educators was estimated to be sufficient to enable the detection of a minimum of 5% change in HIV prevalence in each reporting domain with 80% power at 5% level of significance and assuming a design effect of two due to potential clustering at school level [15].

A multi-stage stratified cluster design with probability proportional to size sampling was used to draw a random sample of schools and all educators. The sample was stratified by all nine provinces and all the 101 educational districts in the country. In each district a sample of schools were randomly selected with probability proportional to size. This implies that schools with a larger number of educators had a higher chance of being selected [15].

In each selected school, all educators present on the day of the survey were eligible to participate in the study. The sample comprised 1380 randomly selected schools which included primary, secondary, combined, and intermediate schools. The study included educators and school management in public schools who were teaching grades R to 12. Participants were employed full-time or part-time with salaries paid either by the state or by school governing bodies [15].

Out of the 1,380 schools sampled, 1,365 were valid and 96.2% agreed to participate in the study. The proportion of non-response was 3.8%, which included refusals (2.6%) and schools not visited or closed down (1.2%). Of these, 16,391 (65.2%) agreed to provide a blood specimen for HIV testing. The proportions of HIV testing non-response included educators who were interviewed but refused to provide a blood sample (20.3%), educators who refused to be interviewed or to provide a blood sample (5.9%), educators who were present at school but not available to participate (8.0%), and educators who were absent from school on the day of data collection (0.6%) [15].

### Study instruments

The study used a questionnaire to collect educators’ socio-demographic and behavioural information. The questionnaire collected information on biographical details, residence and mobility, socio-economic status; information on teaching responsibilities and work load of the educator, impact of HIV on educators; workplace absenteeism, morale and job satisfaction; HIV knowledge and sexual behaviour; health status, and self-reported NCDs.

### Dried blood spot (DBS)

Consenting educators were tested for HIV using DBS. A blood specimen was obtained using a finger-prick method, by spotting a maximum of five circles onto a Whatman grade 903 Guthrie card and sent to the laboratory for HIV testing. The VironostiKa HIV Uniform II Ag/Ab assay (EIA 1) and Roche Elecys HIV 1 Ag/Ab assay (EIA 2), was used to test for HIV antibodies. All specimens were tested using EIA 1 and those that tested positive using EIA 1 were re-tested using EIA 2. For quality assurance purposes 10 percent of the samples that tested HIV-negative using EIA 1 were re-tested using EIA 2. Any samples producing discordant results with the first two EIAs were submitted to a nucleic acid amplification testing (NAAT) (EIA 3) for final interpretation of discordant samples [15].

### Outcome variable

The outcome variable was based on the question “Within the past five years, did a health practitioner (doctor, nurse, etc.) ever tell you that you have any of the following conditions or treated you for such conditions?” diabetes (Yes/No), cancer (Yes/No), high blood pressure (Yes/No), heart disease (Yes/No), stomach ulcers (Yes/No), and lung problems (Yes/No). The responses were then categorized and dichotomised into presence (Yes = 1) and absence (No = 0) of at least one or more NCDs.

### Explanatory variables

This included background characteristics such as sex (male and female), age (18 to 24 years, 25 to 34 years, 35 to 44 years, 45 to 54 years and 55 years and older), race (Black African and other race groups), marital status (married and not married), locality type (urban formal-. planned, urban informal-unplanned, rural formal-commercial farms, and rural informal-tribal areas), position in the school (teacher/educator, senior teacher, head of department, education specialist, deputy principal/principal), Educators absent and unhealthy, number of days absent in 2014 (0–4 days, 5–19 days, 20 days and more).

### Ethical considerations

Ethical approval for the research protocol was provided by the Human Sciences Research Council Research Ethics Committee (REC: 6/21/05/14). Written informed consent was provided by all educators who agreed to participate. All data were collected anonymously. Data were not linked to any individual or school in the reporting and the discussion of the results.

### Statistical analysis

Frequency distribution and percentages were used to summarize the distribution of each NCD and the presence of one or all NCDs was reported by background characteristics of study participants. Pearson chi-square test was used to compare proportional differences in the presence of NCDs by background characteristics. A multivariate logistic regression model using backward stepwise selection method was fitted to determine factors associated with the presence of selected NCDs among HIV positive educators. Probability for removal of variables in the model was set at a p-value of 0.20. Adjusted Odds ratios (aORs) with 95% confidence intervals (CIs) are reported, and p-values ≤ 0.05 were considered statistically significant. Coefficient plots were used to display the results of the final model. All data were analysed using STATA 13.0 (StataCorp, College Station, TX, USA).

## Results

### Prevalence of individual NCDs

**Fig 1:**
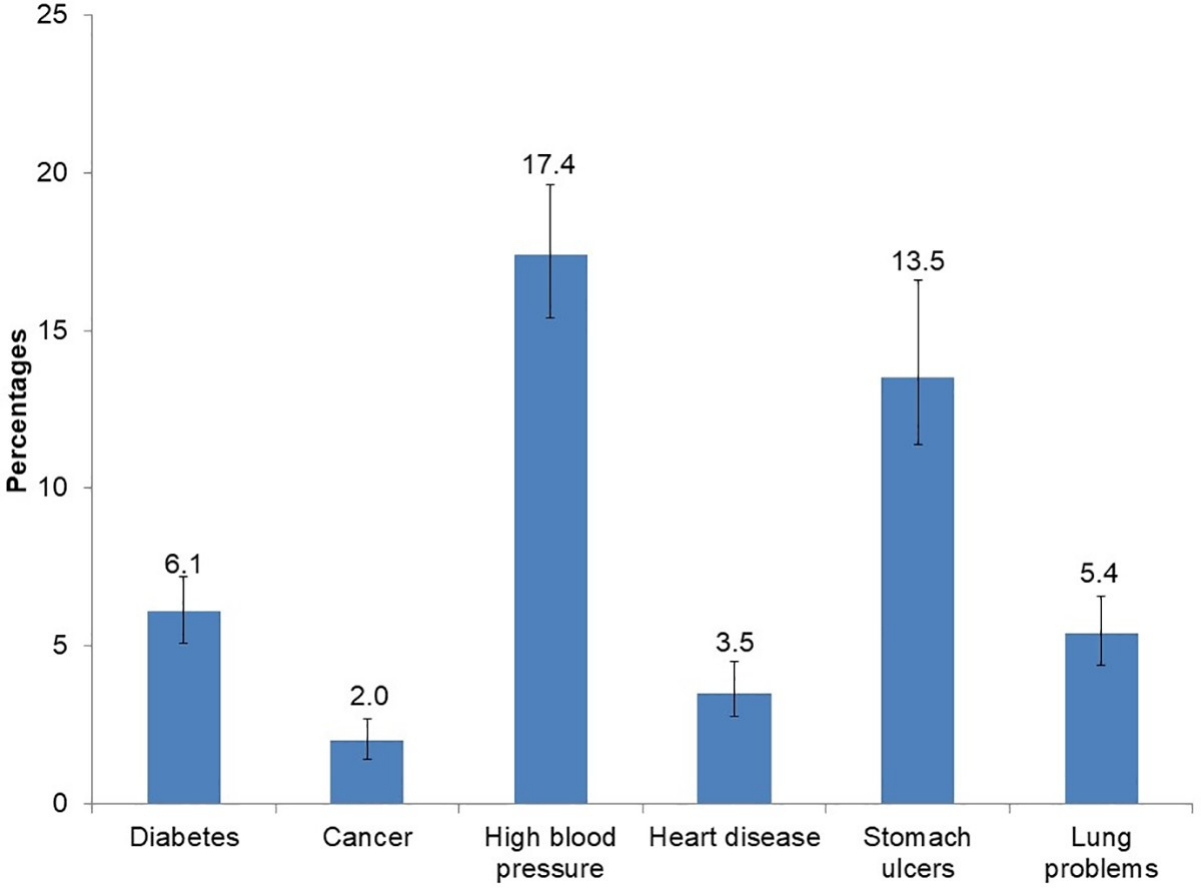
The distribution of non-communicable diseases (NCDs) comorbidities among HIV positive educators in South Africa.

The overall prevalence of self-reported NCDs was 36.9% (95% CI: 34.2–39.6%) out of 2691 HIV positive participants who responded to the NCD questions. Fig 1 shows that of these 64.7% reported no NCD, 26% reported two NCDs and only 2.2% reported three or more NCDs.

**Fig 2:**
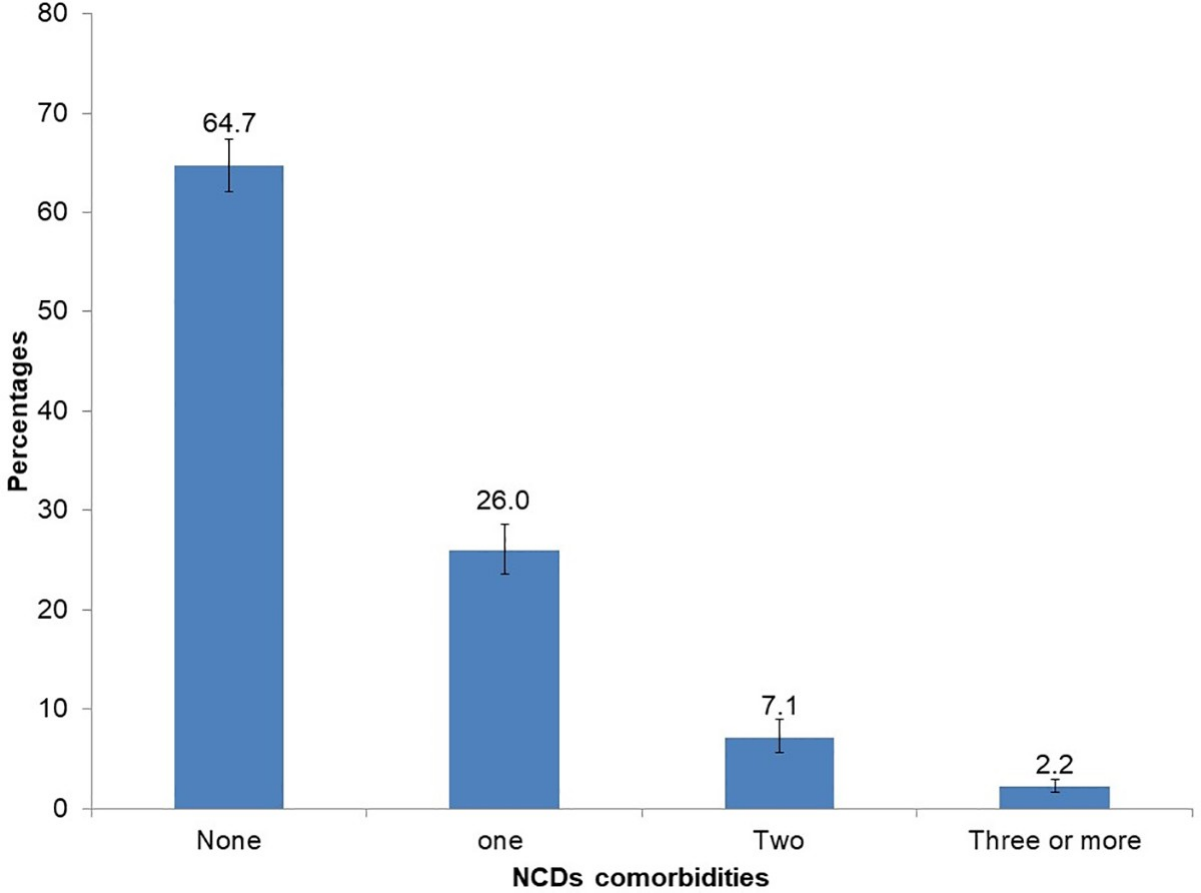
Prevalence of reported non-communicable diseases (NCDs) among HIV positive educators in South Africa.

Fig 2 shows that the commonly reported NCDs were high blood pressure (17.4%) and stomach ulcers (13.5%).

Table 1 shows that the prevalence of diabetes was slightly higher among those 50 years and older (13.1%). The prevalence of high blood pressure was higher among teachers of other race groups (35.2%), those 50 years and older (33.3%) and among teachers in senior positions (26.4%). The prevalence of stomach ulcers were slightly higher among teachers 18–24 years age (22.9%), and those in urban informal schools (21.1%). The prevalence of self-reported Lung / breathing problems, cancers and heart diseases was low.

**Table 1:** Prevalence of self-reported NCDs by background characteristics among HIV positive educators in South Africa.

	Diabetes		Cancer		High blood pressure		Heart disease		Stomach ulcers		Lung problems	
	n	%	N	%	N	%	n	%	n	%	N	%
**Sex**												
Male	598	6.3	598	1.6	598	11.5	598	4.5	598	7.9	599	6.3
Female	2050	6	2054	2.1	2052	19.3	2052	3.2	2053	15.8	2052	5.1
**Age in years**												
18 to 24	29	0	29	0	29	0	29	1.5	29	22.9	29	0
25 to 34	422	1.5	423	1.9	423	7.3	422	2.9	423	10.4	425	3.5
35 to 44	961	3.5	962	2.4	963	15.1	962	2.3	963	17.1	961	5.7
45 to 54	1053	9.1	1055	1.8	1053	21	1054	4.2	1053	12.4	1053	5.9
55+	183	13.1	183	1.4	182	33.3	183	7.8	183	10.8	183	6.9
**Race**												
Black African	2624	6	2628	2	2626	17.2	2626	3.6	2627	13.9	2627	5.4
Other	23	9	23	0	23	35.2	23	0	23	4.6	23	8.9
**Marital status**												
Married	956	7.5	957	2.6	957	18.7	956	4.6	956	12.7	957	6.3
Not Married	1294	4.5	1298	1.6	1297	15.7	1297	2.1	1296	14.5	1296	4
Divorced/Separated	146	7.8	146	3.3	146	22.2	146	8.8	147	15.2	147	7.1
Widower/Widow	249	7.3	248	0.6	247	17.7	248	3.5	249	14.1	248	8.8
**Locality type**												
Urban formal	581	7.2	582	2.7	580	16.5	582	4.4	581	9.4	582	5.9
Urban informal	313	7.2	314	1.9	313	19.8	314	4.4	313	21.1	313	5.5
Rural formal	661	4.6	662	1.1	663	15.8	661	3	661	9.9	661	5.8
Rural informal	989	5.8	990	2.1	990	17.8	990	2.9	993	16.7	992	5.1
**Position in the school**												
Teacher/educator	2191	5.7	2196	2.1	2194	17.2	2194	3.6	2193	14.4	2194	5.3
Senior teacher	75	5.9	76	0.8	76	26.4	76	6.3	76	16.3	76	11.7
Head of department	232	7.9	230	1.6	230	15.5	230	2	232	8.5	231	4.7
Education specialist	9	0	9	0	9	14.6	9	3.5	9	14.8	9	0
Deputy principal/Principal	134	8.8	134	1.2	134	18.6	134	2.8	134	11.3	134	6
**Educators absent and unhealthy**												
Absent	744	6.5	745	2.8	745	16.2	745	4	746	16.1	746	7.2
Present	1882	6	1884	1.7	1882	17.9	1882	3.3	1882	13	1882	4.8
**Number of day absent in 2014**												
0–4 days	535	8.3	535	2.6	536	17.4	535	3.3	536	8.9	534	2.5
5–19 days	1151	5.6	1154	1.6	1154	16.5	1153	3.7	1153	16.1	1152	5.4
20 days and more	167	4.1	168	7.3	167	15.9	168	6.3	168	14.9	169	8.7

### Presence of selected NCDs by background characteristics

Out of a sample of 2691 educators, 26.0% (95% CI: 23.6–28.6) reported one NCD, 7.1% (95% CI: 5.6–9.0) reported two and 2.2% (95% CI: 1.6–2.9) reported more than three NCDs. Table 2 shows that the presence of NCDs was significantly higher among females (40.0%), educators aged 45–54 years (41.1%), those teaching in school located in urban informal areas (48.2%), and those absent 20 days and more (48.8%), respectively.

**Table 2:** The presence of selected non-communicable diseases (NCDs) by background characteristics among HIV positive educators in South Africa.

Variables	N	%	95% CI	p-value
**Sex**				
Male	604	27.4	23.3–31.8	<0.001
Female	2087	40.0	36.8–43.3	
**Age in years**				
18 to 24	30	25.4	10.7–49.1	0.001
25 to 34	434	27.6	22.6–33.2	
35 to 44	973	34.4	29.0–40.3	
45 to 54	1071	41.1	37.4–44.9	
55+	183	47.8	38.9–56.8	
**Race**				
Black African	2666	36.8	34.1–39.6	0.334
Other	23	46.9	27.7–67.1	
**Marital status**				
Married	960	36.8	32.4–41.3	0.790
Widower/Widow	593	36.0	32.6–39.5	
**Locality type**				
Urban formal	586	31.6	27.3–36.4	0.010
Urban informal	316	48.2	37.6–59.0	
Rural formal	666	33.0	29.0–37.3	
Rural informal	999	37.0	33.0–41.3	
**Position in the school**				
Teacher/educator	2208	35.8	32.8–39.0	0.242
Senior teacher	77	51.8	37.7–65.7	
Head of department	235	34.3	27.6–41.7	
Education specialist	9	25.9	5.8–66.2	
Deputy principal/Principal	135	39.1	29.1–50.0	
**Educators absent and unhealthy**				
Yes	750	37.9	33.8–42.1	0.461
No	1897	35.8	32.5–39.3	
**Number of day absent in 2014**				
0–4 days	541	34.5	29.5–39.8	0.021
5–19 days	1159	35.1	31.2–39.2	
20 days and more	191	48.8	39.8–57.9	

### Multivariate model

**Fig 3:**
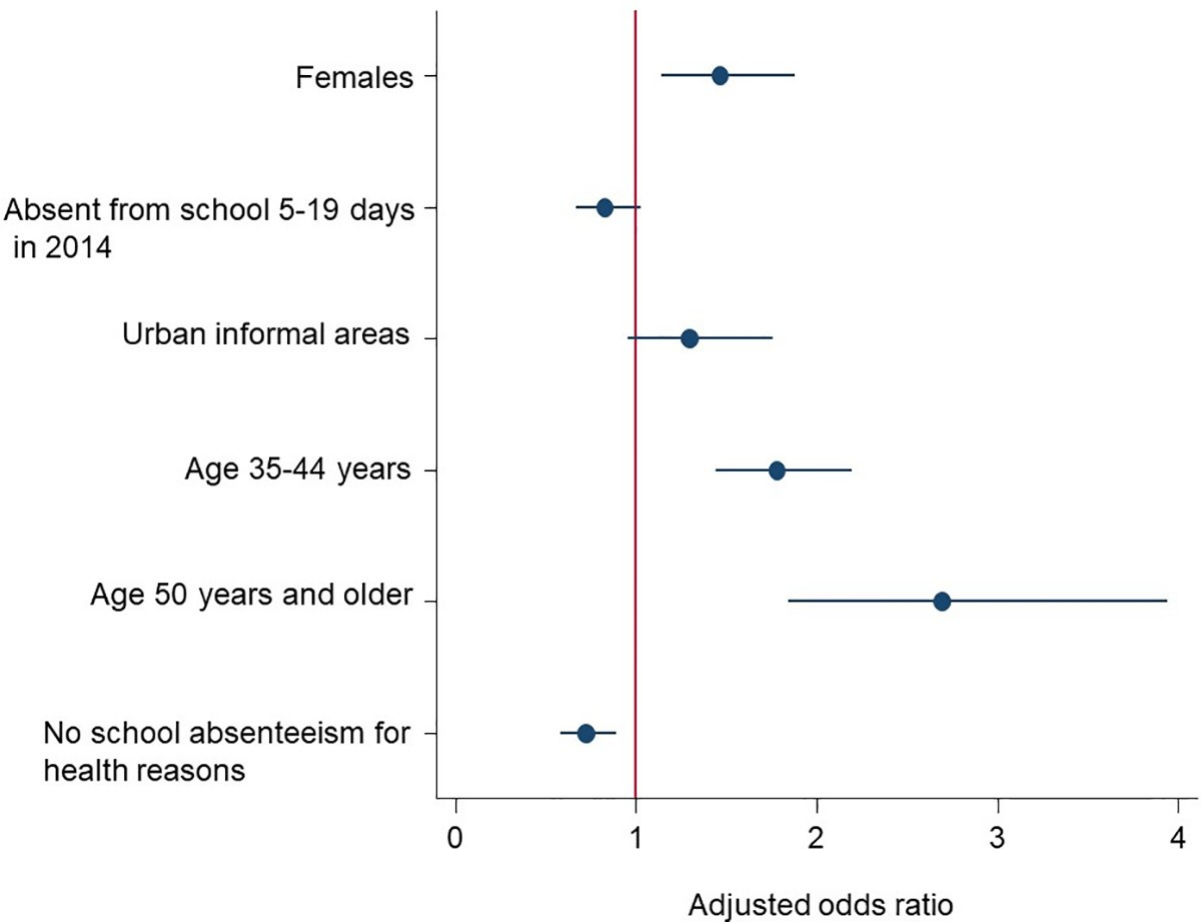
Coefficient plots of multivariate stepwise backward logistic regression model of factors associated with reported non-communicable diseases (NCDs) among HIV positive educators in South Africa.

Fig 3 shows the results of the multivariate stepwise backward logistic regression model between the presence of selected NCDs and explanatory variables. The increased odds of reporting the presence of NCDs was significantly associated with being female than male [aOR = 1.5: 95% CI (1.1–1.9), p<0.002], age 45 to 54 years [aOR = 1.8: 95% CI (1.4–2.2), p<0.001], and 55 years and older than those 18 to 24 years old [aOR = 2.7: 95% CI (1.8–3.9), p<0.001). While the decreased odds of reporting the presence of NCDs was significantly associated with not being absent from school for health reasons [aOR = 0.7: 95% CI (0.6–0.9), p = 0.003].

## Discussion

The study revealed that more than a third of HIV positive educators had NCDs. The more prevalent NCDs were high blood pressure and stomach ulcers. These were also the most commonly reported NCDs in the 2004/5 South African Public Schools Survey [16]. This suggests the intersection of NCDs with HIV has remained unchanged in the past ten years in this population. This linkage highlights the need to integrate responses for HIV and NCDs among educators in this sector.

The findings showed that women were more likely to report NCDs than men. This may be a reflection of the different health seeking behaviour by women when compared to men. Nevertheless, studies have advanced that women's susceptibility to NCDs is linked to physical inactivity and obesity, which are reported to be higher among women than the men [6,7,17,18]. This suggests that successful management of both HIV and NCDs should involve strategic screening for NCDs and promotion of healthy behaviours for all educators in the sector.

The presence of selected NCDs was associated with rise in age. Generally, advanced age is associated with low physical activity and poor health [6,17–20]. Increased access to anti-retroviral treatment has turned HIV in to a chronic disorder. HIV positive people live longer. The increasing age comes with an increased risk of NCD comorbidity [6, 20]. There is therefore a need to control and prevent NCDs and their common risk factors among educators living with HIV.

Similar to current findings in the 2004/5 South African Public Schools Survey HIV and NCDs were observed to contribute to poor health and absenteeism [16]. Given the fact that NCDs in the same way as HIV affects health directly and indirectly [3], both can hinder the ability of educators to perform their duties. Since the joint burden may have major adverse effects on the educator wellbeing and performance. Prevention and management of HIV infection and NCDs through health promotion, treatment and regular monitoring of treatment outcomes is vital for the education sectors.

### Limitations

The prevalence NCDs may be underestimated by self-reporting and not considering other NCDs. Furthermore, it have would have been better to consider each NCD as a single outcome and not combined them. However, we were also limited by the small numbers hence the pooled analysis. There may also be other potential unmeasured risk factors and / or confounders that were not taken into account in this analysis. Furthermore, the cross-sectional nature of the survey does not allow for proper inference of the causality between NCDs and explanatory variables in the context of HIV among educators. Nevertheless, this study provides the evidence base on the prevalence of NCDs and their associated risk factors among HIV positive educators using a nationally representative sample. This is useful in planning and rolling out the NCDs active screening as an integral part of the HIV programmes including interventions such as prevention, treatment and care amongst public school educators in SA.

### Conclusion

The study revealed that the presence of NCDs was relatively high. The commonly reported NCDs were high blood pressure and stomach ulcers. The analysis also identified specific background characteristics as predisposing to NCDs. Furthermore, the findings suggest a connection between absenteeism and presence of NCDs among HIV positive educators. Therefore, regular monitoring of NCD and associated risk factors is of paramount importance among HIV positive educators. There is a need for more research to address the complex interaction between HIV and NCDs in the workforce in order to inform policy and interventions.

## Supporting information

S1 Data Final weighted educator data with NCD HIV ARV. (DTA)
